# Species asynchrony stabilizes productivity over 20 years in Northeast China

**DOI:** 10.1002/ece3.9991

**Published:** 2023-05-03

**Authors:** Bo Jia, Jingyuan He, Xinjie Wang

**Affiliations:** ^1^ Beijing Forestry University Beijing China

**Keywords:** biodiversity–ecosystem functioning, diversity, piecewise structural equation model, species asynchrony

## Abstract

The stability of forest productivity can reflect the functioning of forest ecosystems. It is a crucial topic to understand the relationship between biodiversity and ecosystem functions in ecology. Although previous studies have made great progress in understanding the effects of diversity, species asynchrony, and other factors on community biomass and productivity, few studies have explored how these factors affect the temporal stability of productivity. In this study, we hypothesized that diversity, species asynchrony, and topography would directly or indirectly impact the temporal stability of productivity. To test this hypothesis, we used a multiple regression model and a piecewise structural equation model based on the inventory data over 20 years (5‐year intervals) from 1992 to 2012 at Jingouling Forest Farm in Northeast China. Our results showed that species asynchrony was the main driving factor affecting the temporal stability of productivity. Structural diversity significantly decreased community stability, while species diversity had a nonsignificant effect on it. We found the combination of a multiple regression model and a piecewise structural equation model is an effective method for evaluating the factors that influence community stability. The effect of species asynchrony is crucial for understanding the ecological mechanisms underlying the diversity–stability relationship in mixed forests.

## Graphical abstract

Piecewise structural equation model (pSEM) was utilized to disentangle how diversity, topography, and species asynchrony directly and indirectly impact community stability. Finally, we can find that species asynchrony was the main driving factor of community stability. pSEM can handle non‐normal distributions and hierarchical structures.

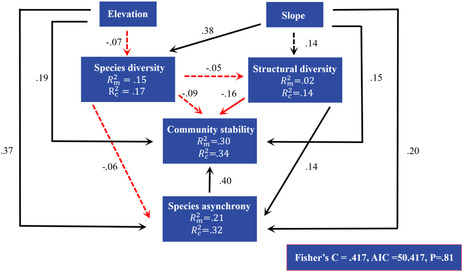

## 1 INTRODUCTION

In the face of global climate change, maintaining ecosystem functions and services has become increasingly essential. In ecology, the temporal stability of ecosystems indicates their ability to consistently provide functions and services amidst environmental disturbances (Ives & Carpenter, 2007; Li et al., 2021; Pennekamp et al., 2018). Forest ecosystem is the largest terrestrial ecosystem of the global land surface (Pan et al., 2013) and plays a vital, irreplaceable role in providing important ecosystem functions and services (Ferreira et al., 2018). The temporal stability of forests, i.e., the constancy of ecological variables such as productivity over time (Pimm, 1984), is crucial for the ecosystem functions and services provided by forests, including nutrient cycling, wood production, carbon sequestration, and water conservation. Therefore, it has been studied by many researchers (del Río et al., 2017; Jourdan et al., 2020).

The temporal stability of a community can be influenced by multiple biotic and abiotic factors (Hautier et al., 2015; Jourdan et al., 2021), exploring what factors affect community stability is always a hot topic in ecology (Valerio et al., 2022). In recent years, studies on community stability have mainly focused on the relationship between diversity and stability, but the results are still controversial (Aussenac et al., 2019; McCann, 2000; Morin et al., 2014; Xu et al., 2021). Empirical evidence suggests that species diversity can increase the temporal stability of forests (Jucker et al., 2014; Schnabel et al., 2019), while some studies have obtained the opposite result (DeClerck et al., 2006; Morin et al., 2014). Although the impact of species diversity on stability has been widely reported, studies on structural diversity are still rare and mainly focus on stand growth (Forrester, 2019; Soares et al., 2016). It is known that structural diversity can affect forest ecosystem functioning (Ali et al., 2016). Pioneering work has shown that promoting species and structural diversity is an effective method for increasing the robustness of forest ecosystem functions (Silva Pedro et al., 2017). Structural diversity, rather than species diversity, has a greater impact on forest productivity (Dănescu et al., 2016). However, empirical studies have also concluded that productivity is not strongly affected by structural diversity (Ali, 2018; Long & Shaw, 2010). This effect of size inequality could differ due to forest types in mixed‐species forests (Forrester & Bauhus, 2016; Liang et al., 2007). In recent years, some studies suggest that variation in tree size plays a key role in the stabilizing effect of diversity (Aussenac et al., 2017; Schnabel et al., 2019).

In addition to the factors mentioned above, the relationship between diversity and stability may also depend on species asynchrony (Sasaki et al., 2019) and environmental conditions (Xu et al., 2015). Species asynchrony refers to the asynchronous dynamics across species in response to fluctuating environmental conditions, which can be explained by niche differentiation (Loreau & de Mazancourt, 2008). It is considered a key mechanism for controlling the stability of natural communities (Craven et al., 2018; Isbell et al., 2009; Lepš et al., 2017; Valencia et al., 2020). In forest ecosystems, the relationship between species asynchrony and stability has been confirmed by many studies (Morin et al., 2014; Schnabel et al., 2021; Yu et al., 2020; Yuan et al., 2019). The insurance hypothesis (Yachi & Loreau, 1999) predicts that diversity stabilizes community productivity through species asynchrony, and a huge amount of evidence has reported a positive relationship between species asynchrony and stability (Ma et al., 2021; Schnabel et al., 2021). However, some studies have found that species asynchrony has a negative impact on the temporal stability of plant communities (Chi et al., 2019; Craven et al., 2018). These ambiguous results demonstrate our limited understanding of the relationship between asynchrony and stability, and further research is necessary to fully reveal this relationship. As an important ecological factor, the topography is an indispensable factor to consider in investigations and is crucial in determining community stability. On the one hand, topography acts as a vital role in shaping the distribution of forest tree species by representing resource availability (Guo et al., 2017). On the other hand, topography determines the geographic gradient of species diversity (Zhang et al., 2019) and indirectly affects forest stability through its impact on diversity (Belote, 2018). It is known that the stability of the ecosystem would change significantly with changes in elevation (Geng et al., 2019), and slope usually promotes community stability (Ouyang et al., 2020). Therefore, it is important to consider these factors to better understand how topographic factors influence stability.

Mixed forests are increasingly recognized for their ability to provide multiple ecological services due to their diverse species compositions, which helps them mitigate and adapt to climate change (Calama et al., 2021), therefore, have attracted more and more attention (Hiura et al., 2019). However, data from long‐term series of mixed forests usually take decades, which lead to the fact that analyses of the temporal stability of communities related to tree diversity in mixed forests are not systematic. Furthermore, most studies have focused on the temporal stability of aboveground biomass rather than productivity due to the limited availability of forest inventory data. To accurately calculate the community temporal stability of forest productivity, at least four repeated forest inventory data are required (Yuan et al., 2019), which has led to a scarcity of research in this area.

In general, a lack of understanding and research on the factors affecting plant community productivity stability, especially in mixed forests, highlights the need for direct observational data from long‐term series in forest ecosystem studies. To address this need, we collected inventory data from 184 permanent sample plots in Northeast China at 5‐year intervals from 1992 to 2012. Then, we conducted a multiple regression model and a piecewise structural equation model (pSEM) to explore the effects of species diversity, structural diversity, topography, and species asynchrony on the temporal stability of forest productivity. In this study, we hypothesized that species diversity, structural diversity, and topography can not only directly impact the temporal stability of the community but also indirectly impact it through species asynchrony. This study is expected to provide a scientific basis for the management of mixed forests in Northeast China in future.

## 2 MATERIALS AND METHODS

### 2.1 Study area

The study was conducted in Jingouling Forest Farm (130°05′–130°20′ E, 43°17′‐43°25′ N) in Northeast China, where the altitude ranges from 653 to 785 m. This area is controlled by a temperate continental monsoon climate, with a mean annual temperature of 3.9°C and an annual precipitation of 600–700 mm. The main soil type is dark brown soil. The dominant tree species in Jingouling Forest Farm are *Betula platyphylla*, *Populus davidiana*, *Acer mono*, *Tilia amurensis*, *Picea jezoensis*, *Pinus koraiensis*, *Abies nephrolepis*, *Korean pine*, etc.

### 2.2 Experimental design and data collection

**FIGURE 1:**
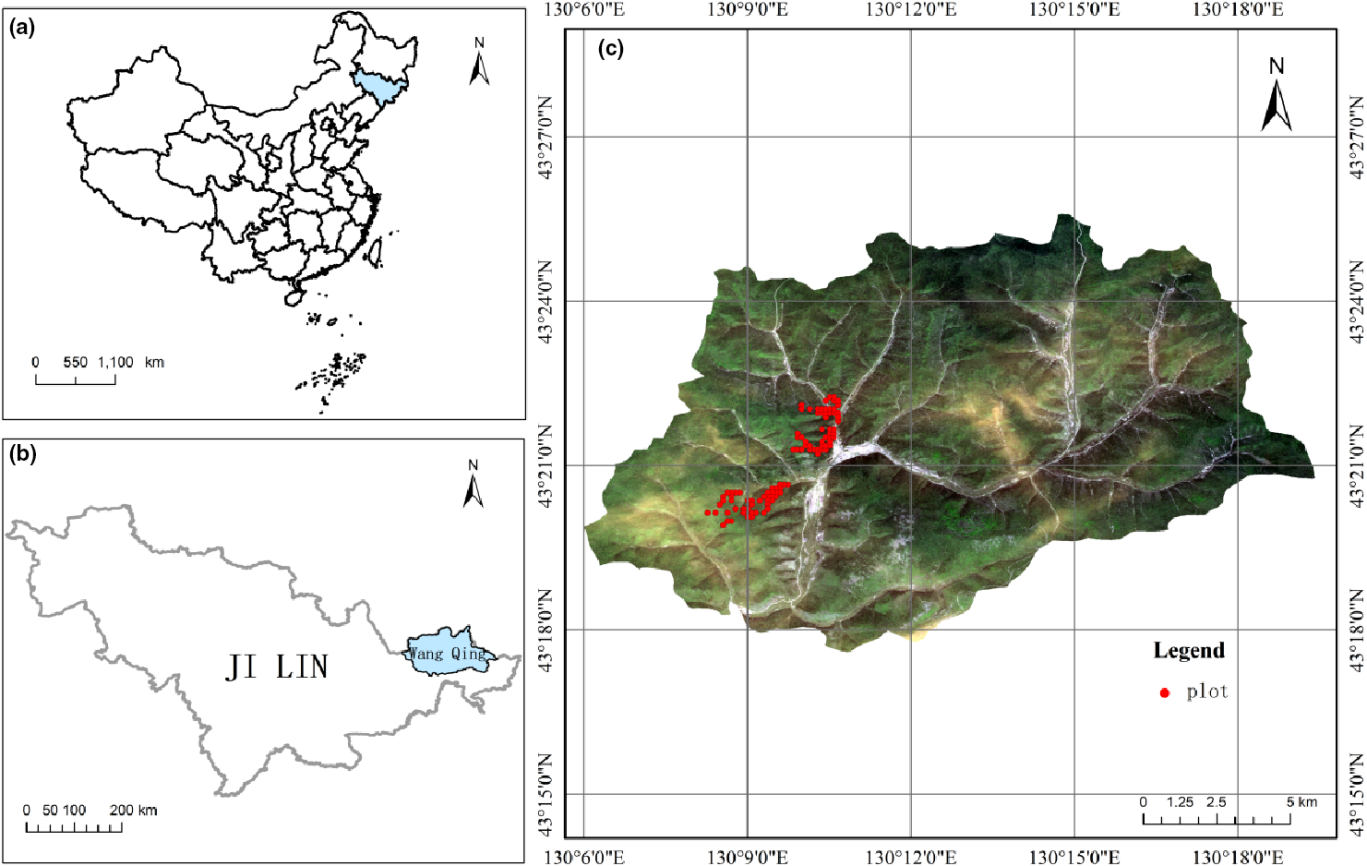
Spatial distribution of permanent plots (a) China (b) Ji Lin province (c) Jingouling forest farm.

In 1987–1988, we selected representative forest land in jingouling forest farm to establish fixed sample plots in order to understand the services and functions of the forest and conduct forest management more effectively. A total of 262 permanent plots were established, the size of each plot is 0.04 ha, which were spaced 90 m apart from each other. Community inventories have been conducted since then from July to August in each year, individual trees with a diameter at breast height (DBH) of at least 5 cm were repeatedly measured and tagged to record information on tree status (dead or alive), location, DBH, and species. In addition to measured data of individual trees, plot level information for elevation (m) was recorded using GPS and slope (°) was measured with a compass. However, 38 plots were gradually abandoned for measurement due to pests and diseases. In this study, there were measurement errors in data and personal errors in records. Therefore, after eliminating the abnormal data, we used data from 184 permanent plots, covering a timespan from 1992 to 2012 at 5‐year intervals. The spatial distribution of the permanent plots is presented in Figure 1.

According to the proportion of tree volume (Moreau et al., 2020), the permanent sample plots were classified into four forest types: Coniferous forests (CF), Coniferous mixed forests (CMF), Broad‐leaved mixed forests (BMF), Coniferous and broad‐leaved mixed forest (CBMF). The forest‐type classification standard was listed in Table 1. The basic information of the sample plots in different forest types was described in Table A1. The volume equations for each tree species of Northeast China were described in Table A2.

**TABLE 1:** Forest type classification standard.

Forest type	Classification standard
Coniferous forest	Single coniferous tree species ≥65% of total volume
Coniferous mixed forest	Coniferous species ≥65% of total volume
Coniferous and broad‐leaved mixed forest	Broad‐leaved or coniferous species account for 35%–65%
Broad‐leaved mixed forest	Broad‐leaved species ≥65% of total volume

### 2.3 The temporal stability of productivity (TS)

In this study, we utilized tree allometric equations (Chen & Zhu, 1989; Wang, 2006) to quantify aboveground biomass (Table A3). Forest productivity was subsequently quantified as aboveground biomass increments between every two adjacent inventories (Ouyang et al., 2019). Here, the temporal stability of productivity within each plot was quantified as the ratio of the mean community productivity (*μ*) and standard deviation of community productivity (*σ*) (Lehman & Tilman, 2000), which reflects the fluctuation of community productivity over the years.

(1) $$\mathrm{TS}=\frac{\mu }{\sigma },$$

where *μ* represents the mean productivity of community, *σ* represents the variance of productivity of community.

### 2.4 Species asynchrony, diversity and topographic variables

Species asynchrony was defined as follows:

(2) $$\text{Species asynchrony}=1-\frac{\delta^{2}}{{\left(\sum_{i=1}^{N}\delta \right)}^{2}},$$

where δ is the interannual variance of community productivity and *i* is the interannual standard deviation of productivity of species *i* in a community with *N* species (Hautier et al., 2014; Jucker et al., 2014).

Here, we selected species diversity and structural diversity indices to enhance our understanding of the processes driving community stability. Species diversity was quantified using the Shannon diversity index (Shannon, 1948), while we used the coefficient of variation in diameter (${\mathrm{CV}}_{\mathrm{DBH}}$), a widely‐used metric for forest diameter diversity, to quantify structural diversity.

(3) $$\text{Shannon}=\sum_{i=1}^{N}P_{i}*\ln\left(P_{i}\right),$$

(4) $${\mathrm{CV}}_{\mathrm{DBH}}=100*\frac{{\mathrm{SD}}_{\mathrm{DBH}}}{{\bar{X}}_{\mathrm{DBH}}},$$

where $P_{i}$ is the proportion of the number of individuals of the *i*th species in each sample plot to the overall, and *N* is the number of species in each plot. ${\bar{X}}_{\mathrm{DBH}}$ and ${\mathrm{SD}}_{\mathrm{DBH}}$ are the mean tree DBH and the Standard deviation of DBH.

Two topographic attributes were used as predictor variables to assess the effects on community stability, i.e., elevation and slope for each plot.

### 2.5 Statistical analyses

We calculate the average values obtained from the five surveys conducted on each plot to represent the overall condition during the entire survey period. Prior to incorporating the data into the model, the explanatory variables were standardized using the Z‐score. To enhance the model construction, we examined the bivariate relationships between community stability and the various factors that impact it.

Next, we conducted a multiple regression model to assess the effects of elevation, slope, species diversity, structural diversity, and species asynchrony on community stability. To probe the mean differences in species asynchrony, diversity, topography and community stability among different forest types, one‐way ANOVAs were utilized, with an alpha significance level set at .05.

We conducted a piecewise structural equation model (pSEM) to further examine the relationship between the driving factors and stability. The pSEM approach extends traditional SEM and can handle non‐normal distributions and hierarchical structures (Lefcheck, 2016). In this study, the impact of forest type on community stability is nonsystematic and unpredictable, but it can explain random changes in fixed factors (Ali et al., 2020; Prado‐Junior et al., 2016). Therefore, we treated forest type as a random effect (i.e., classification variable). All effects were measured using standardized coefficients, with coefficients greater than 0.1 indicating strong effects and coefficients less than 0.1 indicating weak effects (Chou & Bentler, 1995; Schreiber et al., 2006). In the case of over‐fitted or poorly‐fitted models, we employed the direction separation test in the confirmatory path analysis to test whether any missing critical paths should be added or whether any additional paths should be excluded. Fisher's *C* and *p* values were used to assess the goodness of model fit (Shipley, 2013), with a *p* value > .05 indicating that the model structure is reasonable. For each dependent variable in pSEM, we calculated the conditional *R* ^2^ ($R_{c}^{2}$) and marginal *R* ^2^ ($R_{m}^{2}$), which represent the variance explained by fixed (i.e., $R_{m}^{2}$) and random factors (i.e., $R_{c}^{2}$ minus $R_{m}^{2}$) (Lefcheck, 2016).

All the statistical analyses were implemented in R v.4.0.2 (R Core Team, 2021). The indexes of diversity were calculated in “vegan” package (Oksanen et al., 2015), and the piecewise structural equation model was completed in “piecewiseSEM” package (Lefcheck, 2016).

## 3 RESULTS

**FIGURE 2:**
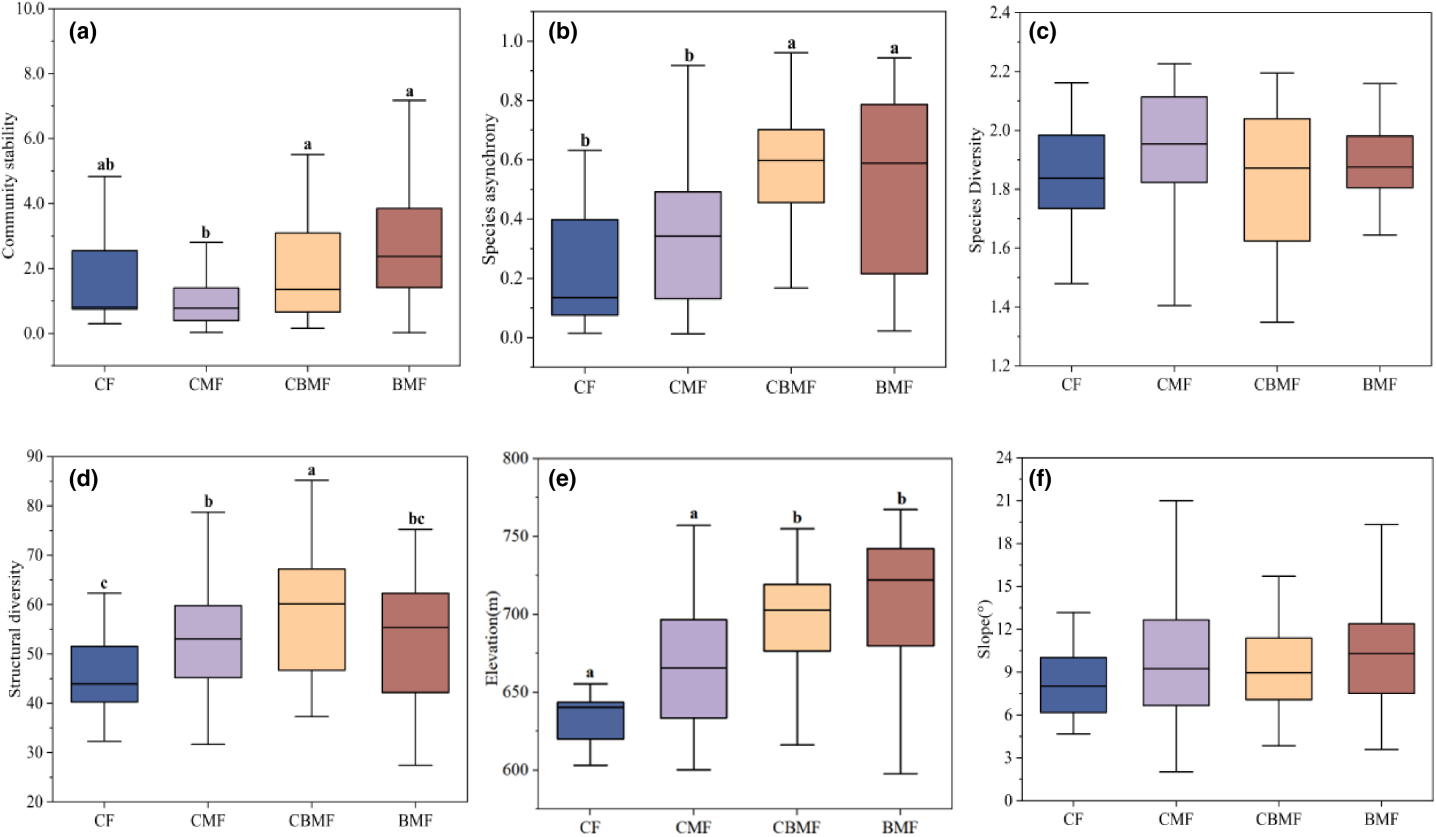
Community stability, species asynchrony, species diversity, structural diversity, and elevation, slope (a–f) of the four types of forests. The different letters at the top of each box represent significant differences between different forest types (*p* < .05). BMF, broad‐leaved mixed forests; CBMF, coniferous and broad‐leaved mixed forests; CF, coniferous forests; CMF, coniferous mixed forests.

There were significant differences in community stability, species asynchrony, structural diversity, and elevation between different forest types, while there was no significant difference in species diversity or slope (Figure 2a–f). Specifically, community stability of CBMF and BMF were significantly higher than that of CMF (CBMF, *p* = .017; BMF, *p* < .001); species asynchrony of CBMF and BMF was significantly higher than that of CF (CBMF, *p* = .000; BMF, *p* = .001) and CMF (CBMF, *p* < .001; BMF, *p* = .002); Structural diversity of CBMF was significantly higher than that of CF (CBMF, *p* = .004); Elevation of CBMF and BMF were significantly higher than that of CF (CBMF, *p* = .042; BMF, *p* = .001) and CMF(CBMF, *p* = .037; BMF, *p* < .001).

**FIGURE 3:**
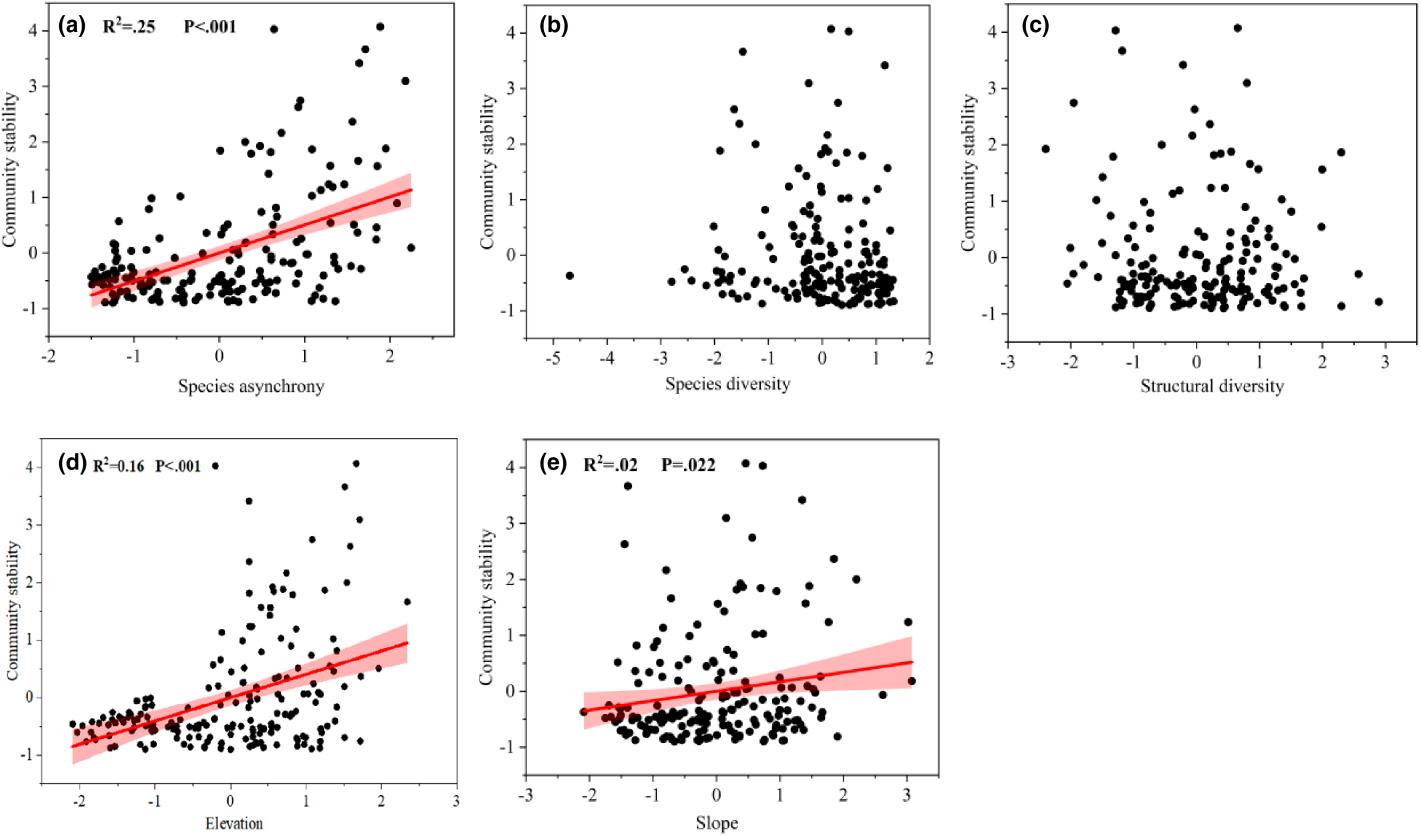
Bivariate relationships between species asynchrony, species diversity, structural diversity, elevation, slope, and community stability (a–e). The black dots correspond to the values of the explanatory variables. The red lines represent significant relationships at *p* < .05, while the relationships without red lines are nonsignificant at *p* > .05. The red shaded areas represent the 95% CI of the model.

We tested the bivariate relationships between community temporal stability and its influencing factors. The results showed species asynchrony (Figure 3a), elevation (Figure 3d), and slope (Figure 3e) were significantly positively related to community temporal stability (*p* < .05). However, the relationship between species diversity (Figure 3b), structural diversity (Figure 3c), and stability was not significant (*p* > .05).

**FIGURE 4:**
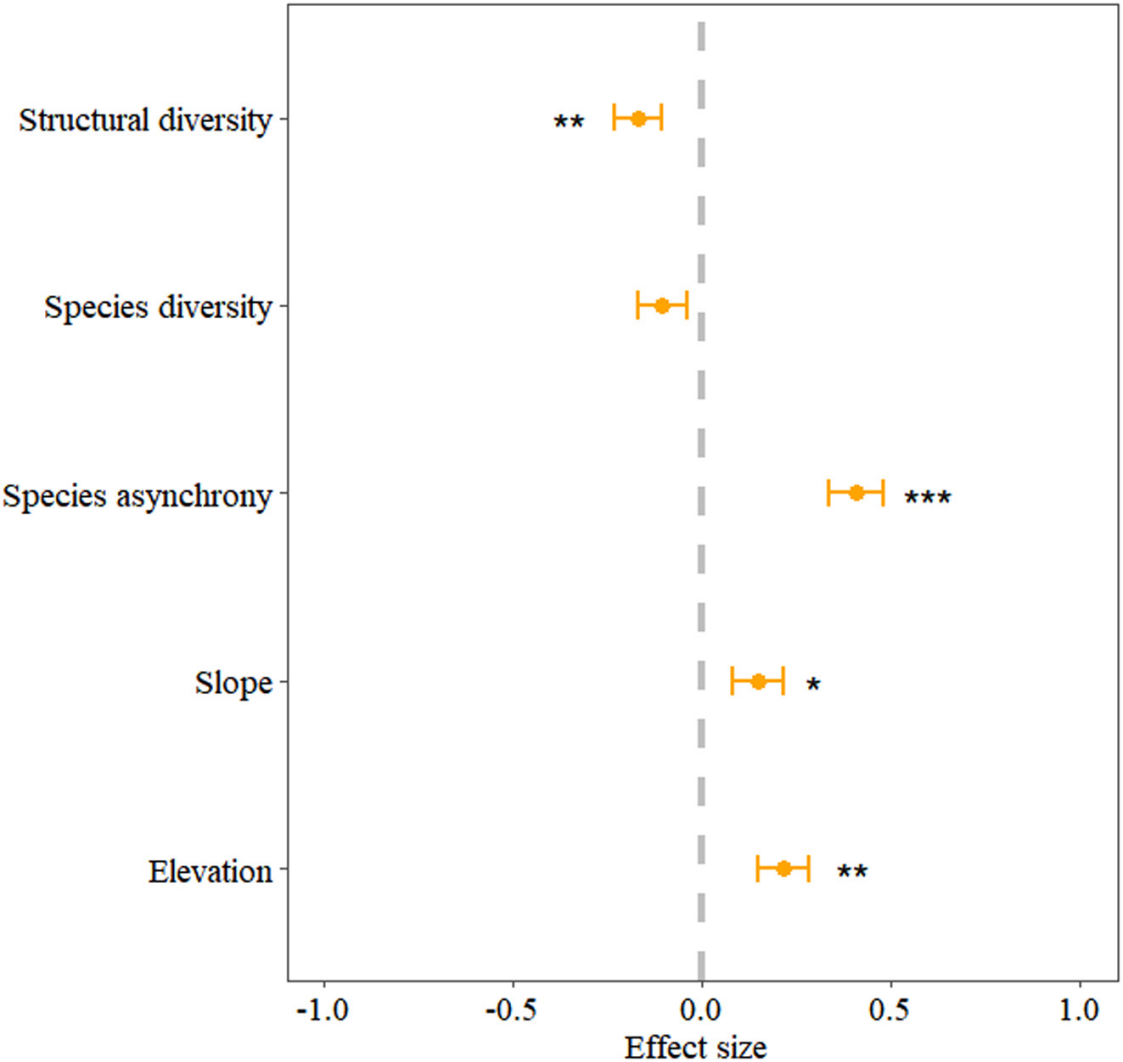
Standardized coefficients of multiple regression model. The yellow points represent the average estimates of the models, and the yellow bars correspond to the 95% CIs. The gray dashed line represents the effect that overlaps with 0 (i.e., neutral). *p < .05, **p < .01, ***p < .001.

Taking into account the interaction between variables, we further used a multiple regression model. The results showed that elevation (β = .22, *p* < .05), slope (β = .15, *p* < .05), and species asynchrony (β = .41, *p* < .05) had significant positive effects on community stability, while structural diversity had a significant negative relationship with it (β = .17, *p* < .05) (Figure 4). Among these factors, species asynchrony accounted for 25.19% of the variation in community stability. Contrary to structural diversity (i.e., CV_DBH_), species diversity (i.e., Shannon index) had aN insignificant impact on community stability (*p* > .05). The summary of the multiple regression model was shown in Table A4.

**FIGURE 5:**
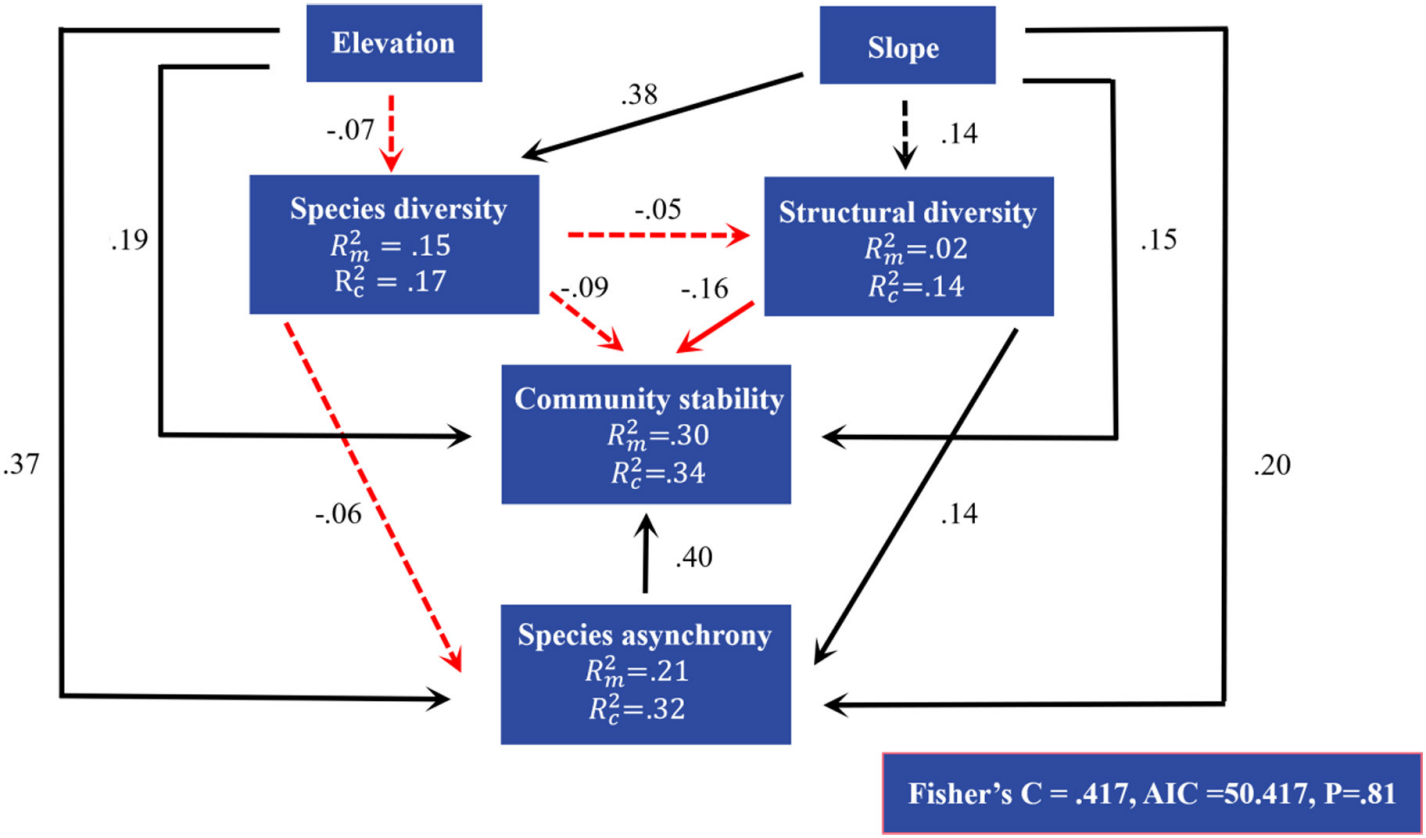
Piecewise structural Equation Model (pSEM) of community stability. The values above lines were standardized coefficients in pSEM. The black and red solid arrows denote pathways that are significant (*p* < .05); The black and red dashed arrows indicate positive and negative insignificantly pathways (*p* > .05).

We concluded that variables explained the marginal and random changes of 30% and 4% of community stability in pSEM, respectively (Figure 5). Species asynchrony (β = .40, *p* < .05), slope (β = .15, *p* < .05), and elevation (β = .19, *p* < .05) were positively related to community stability. Community stability declined with the increase in structural diversity (β = −.16, *p* < .05). Structural diversity (β = .14, *p* < .05), elevation (β = .37, *p* < .05), and slope (β = .20, *p* < .05) were positively related to species asynchrony. Species diversity was positively correlated with slope (β = .38, *p* < .05), while the effect of species diversity on structural diversity was not significant. Species diversity, species asynchrony, and community stability were mainly explained by fixed effects (i.e., its influencing factors); however, structural diversity was mainly explained by random effects (i.e., forest type). The summary of the structural equation model was shown in Table A5.

**FIGURE 6:**
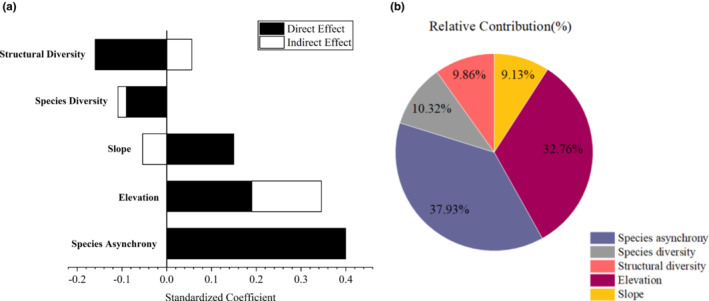
(a) Direct and indirect effects of the influencing factors on the temporal stability of productivity. The black and white bars represent direct and indirect effects, respectively. (b) The pie of relative contributions of the influencing factors, different colors represent the different variables.

In addition to their direct effects, the influencing factors also had indirect effects on the temporal stability of productivity. All variables, except for species asynchrony had both direct and indirect effects on community stability (Figure 6a). Elevation had an indirect impact on community stability through its effects on species diversity and species asynchrony, while slope had an indirect effect on community stability through its impact on species asynchrony and diversity. Both species diversity and structural diversity had an indirect effect on community stability through species asynchrony. While species diversity and structural diversity had an overall negative impact on community stability, the other variables had a positive impact.

To determine the most important factor, we quantified the relative contributions of all variables to community stability by calculating their overall effects (Figure 6b). Species asynchrony explained 37.93% of the variation in community stability, followed by elevation at 32.76%. Compared with diversity factors and topographic factors, species asynchrony was the most critical factor that affects community stability.

## 4 DISCUSSION

This study used repetitively measured inventory data ranging from 1992 to 2012 in Jingouling forest farm, aiming to investigate the relationships between species asynchrony, diversity, topography, and the temporal stability of productivity in natural mixed forests in Northeast China. Our findings showed that species asynchrony, rather than diversity, was the most significant driving factor of community stability, which is consistent with many previous experimental research (Byrnes et al., 2014; del Río et al., 2017). These results underscore the critical role of species asynchrony in determining the temporal stability of productivity, which has been widely validated in various ecosystems (Blüthgen et al., 2016; Jourdan et al., 2021; Muraina et al., 2021). Species asynchrony arises from temporal niche differentiation between species in response to changing environmental conditions (Loreau & de Mazancourt, 2013), which may be caused by external environmental forcing, endogenous species interaction, or a combination of both (Gonzalez & Loreau, 2009).

Inconsistent with previous analyses (del Río et al., 2017; Jucker et al., 2014; Schnabel et al., 2019), we found an insignificant negative correlation between species diversity and community stability. In forest ecosystems, many studies showed that forest productivity would increase with tree species richness (Liang et al., 2016; Zhang et al., 2012). However, as species richness also increases species temporal variability, community stability does not necessarily increase (Schnabel et al., 2021). We also found that structural diversity was negatively related to community stability in pSEM (*p* < .05). Some scholars have stated that productivity always decreases when tree size inequality increases (Edgar & Burk, 2001; Zeller et al., 2018). The negative tree size inequality effect could result both from reduced total stand light interception and reduced light use efficiency, and the effect of tree size inequality on productivity is likely to vary with species shade tolerance (Bourdier et al., 2016). As for the relationship between stability and diversity, it can be affected by complex site characteristics and tree age (Pretzsch, 2005). For example, greater structural diversity appears to have a negative effect on productivity in young stands, but it may have a positive effect in mature stands (Zeller & Pretzsch, 2019). Given that the mixed forests in Jingouling forest farm are uneven‐aged, this could contribute to the negative relationship observed between structural diversity and community stability.

Loreau and de Mazancourt (2013) believed that three main mechanisms were likely to work in the stabilizing effects of biodiversity on ecosystem properties: (1) Asynchronous dynamics across species to fluctuating environments. (2) Species respond to disturbances at different speeds. (3) Reduction in the intensity of species competition. In addition to the above three mechanisms, other mechanisms such as the complementarity effect and selection effect would also play a part in it (Hector et al., 2010). The selection effect proposes that species dominance would lead to higher community productivity, although species dominance would lead to lower species evenness (Loreau & Hector, 2001). However, due to the increase in resource utilization and the promotion and interaction between species, more species can also maintain higher productivity in the community through niche complementarity (Loreau et al., 2001; Tilman et al., 1997).

The stability of forests can be affected by topographic factors, rather than solely on tree diversity (Ouyang et al., 2020). In this study, elevation and slope were both positively correlated with community stability (*p* < .05). Previous studies have demonstrated that elevation was a key topographic factor that significantly impacted species distribution and forest stability (Fu et al., 2019; Wang et al., 2017), while slope may indirectly affect vegetation stability by affecting species composition and diversity (Belote, 2018). Furthermore, areas with higher elevation or steeper slope are less accessible for humans or large animals, therefore are less disturbed (Belote, 2018; Nüchel et al., 2019), which may, improve the temporal stability of community productivity.

Our study provides important evidence for the major role of species asynchrony in stabilizing the productivity of mixed forests and thus provides a useful theoretical basis for developing the management strategies of forest ecosystems in Northeast China. However, there are still some limitations in this study that may have affected the results. Firstly, the mixed forests in our study are uneven‐aged, which makes forecasting dynamics far more complicated. Secondly, we have to admit the narrow selection range of species diversity in this study, which may limit the accuracy of our inference. We believe that the selection of a diversity index in this study may be subjective, which is based on traditional forest survey data. It has low acquisition cost and practical value but may not fully capture the structural characteristics of trees (Dănescu et al., 2016), this could explain the inconsistent results with previous studies. To address these limitations, other diversity indices should be used in future research such as functional diversity indices. Additionally, the role of underground resource availability and resource absorption capacity (Grossiord et al., 2014; Scherer‐Lorenzen, 2014), as well as some abiotic factors such as climate and soil, biotic factors such as tree species composition, wood density and forest age, and socio‐economic factors, should be further examined to understand their impact on the relationship between tree diversity and productivity stability (Ouyang et al., 2020).

## 5 CONCLUSION

In this study, we analyzed the temporal stability of forest productivity using inventory data spanning five censuses over 20 years (1992–2012) in Northeast China. We used a combination of multiple regression models and piecewise structural equation models to analyze the relationships among diversity, species asynchrony, topographic factors, and community stability. Our results demonstrated that structural diversity inhibited the temporal stability of forest productivity during the tree growth period, while species diversity was not significant. It is worth noting that we found species asynchrony was the most critical factor affecting the productivity temporal stability, followed by elevation. The relationship between biodiversity and ecosystem functions (BEF) is always complex, and getting a general conclusion is challenging. More work needs to be conducted to explore this relationship in future.

## AUTHOR CONTRIBUTIONS

**Bo Jia:** Data curation (equal); formal analysis (equal); methodology (lead); validation (equal); writing – original draft (lead); writing – review and editing (equal). **Jingyuan He:** Investigation (equal); writing – review and editing (equal). **Xinjie Wang:** Conceptualization (equal); funding acquisition (lead); methodology (equal); project administration (equal); resources (equal); supervision (equal); writing – review and editing (equal).

## FUNDING INFORMATION

This work was funded by the Key Project of National Key Research and Development Plan [grant number 2017YFC0504101].

## CONFLICT OF INTEREST STATEMENT

The authors declare no conflict of interest.

## Data Availability

The data that support the findings of this study can be accessed on Figshare: https://doi.org/10.6084/m9.figshare.22274635.

## APPENDIX 1

**TABLE A1:** The basic information of the sample plots in different forest types.

Forest type	Variable	Mean	SD	Maximum	Minimum
Coniferous forests (CF), *N* = 13	Community stability	2.00	1.93	6.55	0.30
	Elevation(m)	676.41	47.21	742.10	603.17
	Slope (°)	8.40	2.77	13.18	4.66
	Species asynchrony	0.23	0.20	0.63	0.01
	Shannon Index	1.88	0.18	2.16	1.54
	CV_DBH_ (%)	46.23	6.20	62.33	33.29
Coniferous mixed forests (CMF), *N* = 119	Community stability	1.20	1.35	8.85	0.03
	Elevation(m)	675.58	37.59	757.10	600.24
	Slope (°)	9.77	3.64	21.00	2.03
	Species asynchrony	0.34	0.22	0.92	0.01
	Shannon Index	1.92	0.26	2.23	0.71
	CV_DBH_ (%)	53.26	9.72	78.70	31.69
Broad‐leaved mixed forests (BMF), *N* = 22	Community stability	2.88	2.31	8.20	0.02
	Elevation (m)	713.45	43.63	767.27	597.79
	Slope (°)	10.57	4.38	20.79	3.60
	Species asynchrony	0.53	0.30	0.94	0.02
	Shannon Index	1.82	0.22	2.16	1.28
	CV_DBH_ (%)	53.12	12.68	75.24	27.43
Coniferous and broad‐leaved mixed forest (CBMF), *N* = 30	Community stability	2.24	2.25	8.92	0.16
	Elevation(m)	706.93	38.35	783.09	605.18
	Slope (°)	9.35	3.58	15.72	3.85
	Species asynchrony	0.57	0.23	0.96	0.07
	Shannon Index	1.83	0.27	2.19	1.35
	CV_DBH_ (%)	58.50	12.85	85.19	37.28

**TABLE A2:** Volume equations for each tree species of Northeast China.

Volume	$V=a*D^{b}*H^{C}$
Height	$H=h_{1}-\frac{h_{2}}{D+h_{3}}$

Species	Name	Parameters					
		*a*	*b*	*c*	*h*_1_	*h*_2_	*h*_3_
Larch	*Larix olgensis*	8.47 × 10^−5^	1.97	0.75	34.59	650.53	18.0
Conifer	*Pinus koraiensis*	7.62 × 10^−5^	1.90	0.86	21.84	309.16	14.0
	*Abies nephrolepis*	5.79 × 10^−5^	1.89	0.99	46.40	2137.92	47.0
	*Picea jezoensis*	5.79 × 10^−5^	1.89	0.99	46.40	2137.92	47.0
Hardwood	*Acer momo*	4.88 × 10^−5^	1.84	1.05	24.82	402.09	16.3
	*Fraxinus mandshurica*	5.33 × 10^−5^	1.88	1.00	29.44	468.93	15.7
	*Phellodendron amurense*	5.33 × 10^−5^	1.88	1.00	29.44	468.93	15.7
	*Betula platyphylla*	5.33 × 10^−5^	1.88	1.00	29.44	468.93	15.7
	*Tilia amurensis*	5.33 × 10^−5^	1.88	1.00	29.44	468.93	15.7
	*Betula costata*	5.33 × 10^−5^	1.88	1.00	29.44	468.93	15.7
	*Populus ussuriensis*	5.33 × 10^−5^	1.88	1.00	29.44	468.93	15.7
	*Ulmus pumila*	5.33 × 10^−5^	1.88	1.00	29.44	468.93	15.7

*Note*: *D*, *H*, and *V* refer to tree diameter at breast height, tree height, and volume, respectively. *h*_1_, *h*_2_, and *h*_3_ are parameters.

**TABLE A3:** Allometric equations for the calculation of aboveground biomass for each tree species in Northeast China.

Species	Equations	References
*Abies holophylla*	AGB = 1000 × 0.0737 × (DBH)^2.51264^	Chen and Zhu (1989)
*Abies nephrolepis*	AGB = 1000 × 0.0737 × (DBH)^2.51264^	Chen and Zhu (1989)
*Acer pictum* subsp. *mono*	AGB = 10^(1.930 + 2.535 × log10(DBH))^	Wang (2006)
*Betula platyphylla*	AGB = 10^(2.159 + 2.367 × log10(DBH))^	Wang (2006)
*Betula costata*	AGB = 10^(2.214 + 2.400 × log10(DBH))^	Wang (2006)
*Carpinus cordata*	AGB = 1000 × 0.09802 × (DBH)^2.2993^	Chen and Zhu (1989)
*Fraxinus chinensis* subsp. *rhynchophylla*	AGB = 10^(2.213 + 2.417 × log10(DBH))^	Wang (2006)
*Fraxinus mandshurica*	AGB = 10^(2.216 + 2.408 × log10(DBH))^	Wang (2006)
*Juglans mandshurica*	AGB = 10^(2.235 + 2.287 × log10(DBH))^	Wang (2006)
*Larix gmelinii*	AGB = 10^(1.997 + 2.451 × log10(DBH))^	Wang (2006)
*Maackia amurensis*	AGB = 1000 × 0.0737 × (DBH)^2.51264^	Chen and Zhu (1989)
*Phellodendron amurense*	AGB = 10^(1.942 + 2.232 × log10(DBH))^	Wang (2006)
*Picea jezoensis* var*. komarovii*	AGB = 1000 × 0.0744 × (DBH)^2.5411^	Chen and Zhu (1989)
*Picea koraiensis*	AGB = 1000 × 0.0744 × (DBH)^2.5411^	Chen and Zhu (1989)
*Pinus koraiensis*	AGB = 10 ^(2.236 + 2.144 × log10(DBH))^	Wang (2006)
*Populus davidiana*	AGB = 10^(1.826 + 2.558 × log10(DBH))^	Wang (2006)
*Quercus mongolica*	AGB = 10^(2.002 + 2.456 × log10(DBH))^	Wang (2006)
*Taxus cuspidata*	AGB = 1000 × 0.0737 × (DBH)^2.51264^	Chen and Zhu (1989)
*Tilia amurensis*	AGB = 10^(1.606 + 2.668 × log10(DBH))^	Wang (2006)
*Tilia mandshurica*	AGB = 10^(1.606 + 2.668 × log10(DBH))^	Wang (2006)
*Ulmus davidiana*	AGB = 1000 × 0.09802 × (DBH)^2.2993^	Chen and Zhu (1989)
*Ulmus laciniata*	AGB = 1000 × 0.09802 × (DBH)^2.2993^	Chen and Zhu (1989)
*Ulmus macrocarpa*	AGB = 1000 × 0.09802 × (DBH)^2.2993^	Chen and Zhu (1989)
Other species	AGB = 10^(1.826 + 2.558 × log10(DBH))^	Chen and Zhu (1989)

Abbreviations: AGB, aboveground biomass; DBH, diameter at breast height.

**TABLE A4:** The summary of the multiple regression model.

Index	Estimate	*SE*	*t* Value	*p* Value
Species asynchrony	0.41***	0.07	5.68	<.001
Structural diversity	−0.17**	0.06	−2.68	.01
Species diversity	−0.10	0.07	−1.57	.12
Elevation	0.22**	0.07	3.12	<.01
Slope	0.15*	0.07	2.18	.03

**p* < .05, ***p* < .01, ****p* < .001.

**TABLE A5:** Piecewise structural equation model for the effects of species asynchrony, species diversity, structural diversity, elevation, and slope on community stability.

Response	Predictor	Std.Estimate	*SE*	*p* Value
Community stability	Species asynchrony	0.40	0.07	<.01
Community stability	Species diversity	−0.09	0.07	.17
Community stability	Structural diversity	−0.16	0.06	.01
Community stability	Elevation	0.19	0.07	<.01
Community stability	Slope	0.15	0.07	.03
Species asynchrony	Structural diversity	0.14	0.06	.03
Species asynchrony	Species diversity	−0.06	0.07	.36
Species asynchrony	Elevation	0.37	0.07	<.01
Species asynchrony	Slope	0.20	0.07	<.01
Species diversity	Slope	0.38	0.07	<.01
Species diversity	Elevation	−0.07	0.07	.33
Structural diversity	Slope	0.16	0.08	.04
Structural diversity	Species diversity	−0.05	0.08	.54
